# Development and validation of a nomogram for predicting the risk of developing gastric cancer based on a questionnaire: a cross–sectional study

**DOI:** 10.3389/fonc.2024.1351967

**Published:** 2024-11-11

**Authors:** Zhangsen Huang, Songyao Chen, Songcheng Yin, Zhaowen Shi, Liang Gu, Liang Li, Haofan Yin, Zhijian Huang, Bo Li, Xin Chen, Yilin Yang, Zhengli Wang, Hai Li, Changhua Zhang, Yulong He

**Affiliations:** ^1^ Digestive Medicine Center, The Seventh Affiliated Hospital of Sun Yat-sen University, Shenzhen, China; ^2^ Guangdong Provincial Key Laboratory of Digestive Cancer Research, Digestive Diseases Center, The Seventh Affiliated Hospital of Sun Yat-Sen University, Shenzhen, Guangdong, China; ^3^ General Surgery, Fengqing People’s Hospital, Lincang, China

**Keywords:** gastric cancer, nomogram, risk factor, cancer screening, questionnaire, prediction model

## Abstract

**Background:**

Detection of gastric cancer (GC) at early stages is an effective strategy for decreasing mortality. This study aimed to construct a prediction nomogram based on a questionnaire to assess the risk of developing GC.

**Methods:**

Our study comprised a total of 4379 participants (2326 participants from outpatient at Fengqing People’s Hospital were considered for model development and internal validation, and 2053 participants from outpatients at the endoscopy center at the Seventh Affiliated Hospital of Sun Yat-Sen University were considered for independent external validation) and gastric mucosa status was determined by endoscopy and biopsies. The eligible participants in development cohort from Fengqing people’s Hospital were randomly separated into a training cohort (n=1629, 70.0%) and an internal validation cohort (n=697, 30.0%). The relevant features were selected by a least absolute shrinkage and selection operator (LASSO), and the ensuing features were evaluated through multivariable logistic regression analysis. Subsequently, the variables were selected to construct a prediction nomogram. The discriminative ability and predictive accuracy of the nomogram were evaluated by the C-index and calibration plot, respectively. Decision curve analysis (DCA) curves were used for the assessment of clinical benefit of the model. This model was developed to estimate the risk of developing neoplastic lesions according to the “transparent reporting of a multivariable prediction model for individual prognosis or diagnosis” (TRIPOD) statement.

**Results:**

Six variables, including age, sex, alcohol consumption, cigarette smoking, education level, and Hp infection status, were independent risk factors for the development of neoplastic lesions. Thus, these variables were incorporated into the final nomogram. The AUC of the nomogram were 0.701, 0.657 and 0.699 in the training, internal validation, and external validation cohorts, respectively. The calibration curve showed that the nomogram was in good agreement with the observed outcomes. Compared to treatment of all patients or none, our nomogram showed a notably higher clinical benefit.

**Conclusion:**

This nomogram proved to be a convenient, cost-effective tool to effectively predict an individual’s risk of developing neoplastic lesions, and it can act as a prescreening tool before gastroscopy.

## Introduction

Gastric cancer (GC) is a common malignancy worldwide, ranking fifth in terms of both incidence and cancer-related deaths among malignancies . In 2022, there were over 968,000 new cases and 659,000 deaths globally (1). In China, GC ranks fifth in incidence and third in mortality among malignancies, accounting for 37% of the global incidence and 39.4% of the global mortality (2). The high incidence and mortality rates of GC have posed an enormous burden on China’s healthcare system. The high mortality rate is largely due to low early detection rate, and thus, early detection and treatment play a pivotal role in reducing GC-related deaths. When the diagnosis is made at an early stage, the surgical treatment results in the 5-year survival rate can exceed 90%, even achieving complete remission (3). Indeed, the National Upper Gastrointestinal Cancer Early Detection program, composing GC and esophageal cancer screening program, has achieved remarkable progress in the early detection of GC in China (4), the 5-year survival rate for GC ranges from 35% to 44% in China (3, 5). Fengqing County, located in the southwest region of Yunnan Province, has a high incidence of GC and related mortality. Based on data obtained from Fengqing’s Centers for Disease Control and Prevention, GC is the first most common cancer in Fengqing County, and the 5-year survival rate is only 25.7%, which is lower than the national average survival rate (**Supplementary Figure S1**). Thus, it is imperative to prioritize prevention and timely diagnosis to improve the prognosis of GC.

Currently, upper gastrointestinal endoscopic examination serves as the gold standard for screening and diagnosing GC. However, gastroscopy-based screening is inefficient and impracticable due to the large population that needs screening in China. Hence, a risk stratification method is required as a prescreening tool before gastroscopy. Recently, a risk scoring system was proposed to identify high-risk individuals for GC, who will be subject to endoscopic screening (6). This system incorporates seven predictors, including pickled food consumption, fried food consumption, age, sex, gastrin-17 (G-17), pepsinogen I/II ratio (PGR), and *Helicobacter pylori* (*H. pylori*, Hp) infection (6). This risk scoring system can identify 69.6% of GC in an external validation cohort. However, including serum indicators predictors would substantially increase the cost of risk evaluation and restrict its generalizability, especially in economically disadvantaged regions such as Yunnan Province. Hence, there is an urgent need to establish a non-invasive, simple-to-use, and accurate risk prediction model that can act as a prescreening tool for identifying individuals with a high risk of GC. Such a model would aid in deciding whether endoscopic examination is necessary.

GC is typically a multifactorial and multistep pathological progression. It starts with a series of gastric mucosal changes in the normal mucosa, which progress into non-atrophic gastritis (no-CAG), chronic atrophic gastritis (CAG), intestinal metaplasia (IM), gastric intraepithelial neoplasia (GIN) and ultimately carcinoma (as proposed by Correa’s hypothesis) (7, 8). According to Japanese data, the 5-year cumulative GC incidence ranges from 1.9% to 10% in CAG and from 5.3% to 9.8% in IM (9). Furthermore, the risk of GC occurrence in cases of biopsy-detected GIN are reportedly to be 2.8% to 11.5% for low-grade intraepithelial neoplasia (LGIN) and 10% to 68.8% for high-grade intraepithelial neoplasia (HGIN) (10–13). Thus, enhancing the diagnosis rate of GIN can improve the prognosis. The stage of the GIN is crucial in the progression of GC, as it offers the best chance to avoid GC development. Identification and administration of appropriate intervention or treatment to patients with GIN can reduce the incidence of GC and improve patients’ outcomes.

Nomogram, which is a visual graphic tool that calculates each individual’s probability of clinical events considering the preweight value of each factor, has been widely used to predict the risk and prognosis of various cancers in recent years (14, 15). In this study, we aimed to establish and evaluate a convenient and practical prediction model to assess the risk of developing neoplastic lesions, including GIN and carcinoma. And this model aims to identify a subgroup of individuals at high risk of GC among Chinese population for further diagnostic gastroscopy.

## Materials and methods

### Ethics approval

The study was approved by the Ethics Committee of Fengqing People’s Hospital and the Seventh Affiliated Hospital of Sun Yat-Sen University (KY-2021-105-01), and had been performed in line with the ethical standards in the Declaration of Helsinki. Written informed consents were obtained from all participants in this study.

### Study population

**Figure 1:**
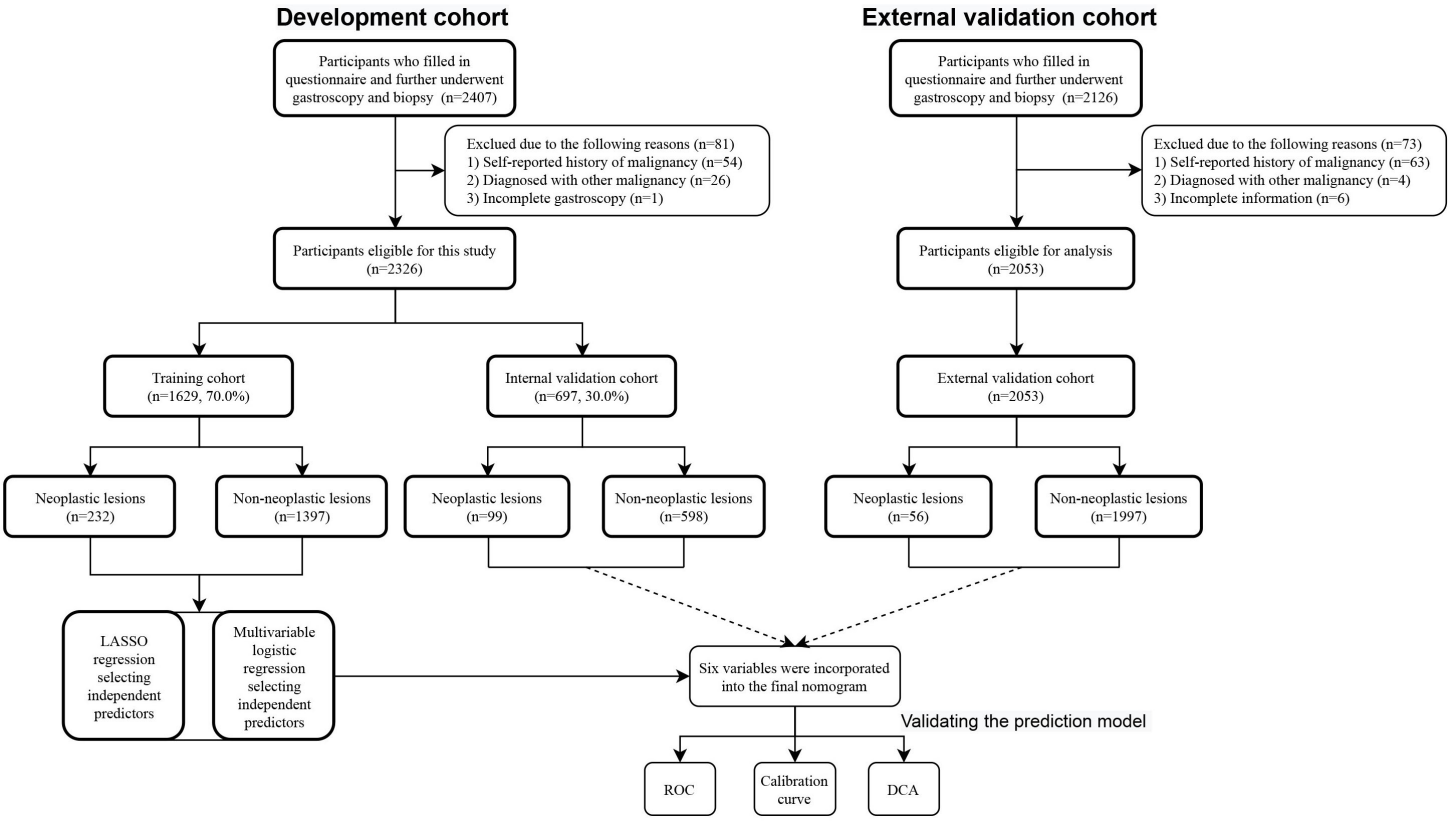
Flow chart of the study population.

A total of 4379 participants underwent upper gastrointestinal endoscopic examination at the endoscopy center at Fengqing people’s Hospital and the Seventh Affiliated Hospital of Sun Yat-Sen University were included in this study between September 2019 and March 2024, 2326 participants were from outpatients at the endoscopy center at Fengqing people’s Hospital and 2053 participants were from outpatients at the endoscopy center at the Seventh Affiliated Hospital of Sun Yat-Sen University. Participants in the development cohort from Fengqing people’s Hospital were randomly divided into a developing set [n=1629, 70.0%] and an internal validation set [n=697, 30.0%], the participants from outpatients at the endoscopy center at the Seventh Affiliated Hospital of Sun Yat-Sen University were used for external validation [n=2053] (**Figure 1**). All participants had completed of upper gastrointestinal endoscopy with valid examination results and were reported as normal gastric mucosa, no-CAG, CAG, IM, LGIN, and HGIN, or carcinoma. These participants were further divided into two groups: non-neoplastic lesions (normal gastric mucosa, no-CAG, CAG, and IM) and neoplastic lesions (LGIN, HGIN, and carcinoma) according to whether the lesion is negative for neoplasia.

### Inclusion and exclusion criteria

The eligibility requirements were as follows: 1) Aged between 35 and 85 years; 2) No history of cancer, mental disorders, or any contraindication to endoscopy; and 3) Had completed of upper gastrointestinal endoscopy with valid examination results. Participants who with a history of cancer were excluded from this study. In addition, we excluded participants who were diagnosed with other malignancies or incomplete information.

The lower age limit of 35 years was used in present study in accordance with the criteria established by the Wuwei Cohort (16), and identifying individuals with GIN at an earlier age could facilitate the implementation of effective preventive strategies. There is no upper age limit in GC screening programs in Japan (17, 18). The upper age limit of 85 years was used in present study as evidenced by data indicating that the incidence rate among individuals aged 75–84 years accounts for more than one-fifth (75,724/358,672) in China (2).

### Data collection

A prevalidated self-report questionnaire was used in this study, and the interviews were conducted face-to-face by highly trained investigators. All variables were questionnaire-based predictors that met any one of the following criteria: 1) biologically plausible in predicting GC risk or 2) reported in at least two published risk prediction studies. The questionnaire included baseline demographic information (age, sex, ethnic group, sleep quality [sleep quality defined as satisfaction with one's sleep experience, including bedtime, times of wake up in the night, frequency difficulty falling asleep, frequency difficulty getting back to sleep after waking up, and frequency wake up feeling tired and worn out, according to a published paper (19)], education, occupation, marital status, and household income, body weight and height), lifestyle (cigarette smoking [smoking more than one cigarette every day for more than 1 year or ever smoked], alcohol consumption [drinking any type of alcohol more than once every week lasting for more than 1 year or ever drank], dietary habits (hot food [hot food means that the temperature of food is over 70 °C], pickled food, fried food, smoked food, spicy food, overnight leftovers, mealtime [mealtime regular was defined as regular time for meals across days and regular meal frequency], red meat, green vegetables and fresh fruit [frequency was defined as more than 3 times/week, frequent fruits and vegetables frequency was defined as more than 3 times/week or >2500 g/week for vegetables and >1250 g/week for fruits], medical history (painkiller, antiplatelet agents, chronic gastritis, gastric ulcer, gastric polyp, hypertrophic gastritis, gastric resection, hyperlipemia, hypertension, diabetes, and pernicious anemia), and family history of GC (FHGC) in first-degree relatives. All endoscopic examinations were performed by well-trained endoscopy doctors at Fengqing People’s Hospital and the Seventh Affiliated Hospital of Sun Yat-Sen University. Pathologists then independently read the biopsy slides and made pathological diagnosis of the biopsy specimens. Family income in ten thousand Yuan and it then was categorized into two groups: low (<5), and high (≥5).

### Nomogram construction

To minimize the potential variables, a least absolute shrinkage and selection operator (LASSO) regression was used in this study. The optimal penalization coefficient (lambda) in the LASSO model was identified by 10-fold cross-validation on the training cohort via minimum criterion. With larger penalties, the estimates of weaker factors shrink towards zero, leaving only the strongest predictors in the model. In the present study, the predictive covariates were selected by the minimum (λ min). These variables identified by LASSO regression analysis were selected for further analysis. Multivariable analyses were performed to determine the independent predictors (p < 0.05), and the nomogram was developed with the selected independent predictors. Each independent risk factor in the nomogram was assigned a score, and the total score was calculated from the participant data to predict the probability of developing GC. The LASSO regression was performed by using "glmnet" package in RStudio software, and the risk prediction nomogram for developing GC was performed using the "rms" package in RStudio software. The model development, internal-external validation, and reporting were performed following the “transparent reporting of a multivariable prediction model for individual prognosis or diagnosis” (TRIPOD) guidelines (20).

### Statistical analysis

Continuous variables were presented as the mean ± standard deviation (SD). Categorical variables were expressed as frequencies. Differences between the two groups for continuous variables were analyzed by the student’s test or Mann-Whitney U test, and the Pearson’s Chi-Square (χ2) test was used for categorical variables. The independent predictors for the development of GC were assessed by multivariable logistic regression analyses. A nomogram was then formulated using the rms package in RStudio software, based on the results of the multivariable logistic regression analyses. The discrimination power of the nomogram was assessed by Harrell’s concordance index (C-index). A calibration plot was used to describe the consistency between the predicted and the actual probability of the occurrence of neoplastic lesions. A decision curve analysis (DCA) graph was generated to evaluate the clinical effectiveness of the model. All statistical analyses were performed using RStudio software (version 4.2.1). Statistically significant in all statistical analyses was defined as p <0.05 in a two-tailed test.

## Results

### Participant’s characteristics

A total of 4379 participants underwent upper gastrointestinal endoscopic examination at the endoscopy center were included in this study between September 2019 and March 2024, 2326 participants were from outpatients at the endoscopy center at Fengqing people’s Hospital and 2053 participants were from outpatients at the endoscopy center at the Seventh Affiliated Hospital of Sun Yat-Sen University. A total of 2326 participants in the development cohort, including1690 cases of no-CAG, 132 cases of CAG, 173 cases of IM, 211 cases of GIN and 120 cases of GC, were recruited in the study, and the participants were divided into two groups: non-neoplastic lesions (n=1995; 1690 participants with normal gastric mucosa or no-CAG, 132 participants with CAG, and 173 participants with IM) and neoplastic lesions (n=331; 211 participants with GIN and 120 participants with GC). A total of 2053 participants, including 1488 cases of no-CAG, 80 cases of CAG, 429 cases of IM, 17 cases of GIN and 39 cases of GC, were recruited from November 2021 to March 2024 as the external validation cohort. A flow chart showing the process of including participants in the study is depicted in **Figure 1**. In the development cohort, there were 1160 males (49.9%) and 1166 females (50.1%), 1753 participants were the Han nationality (75.4%) and 573 participants were minority nationality (24.6%), and the mean age was 55.57 ± 9.06 years (range 35–84 years). In the external validation cohort, there were 1090 males (53.1%) and 963females (46.9%), 2040 participants were the Han nationality (99.4%) and 13 participants were minority nationality (0.6%), and the mean age was 53.21 ± 9.40 years (range 38–85 years). All data of participants, including baseline information, lifestyle, medical history and FHGC, in these two groups are shown in **Table 1**. The baseline demographic and clinicopathologic characteristics of the development cohort are shown in **Table 2**, and none of the variables differed significantly between the training and internal validation cohort in all characteristics (all p > 0.05), indicating reasonable grouping of participants.

**Table 1:** Characteristics of the participants who enrolled in our study.

Variables*	Training Cohort (n = 1629)			Internal Validation Cohort (n = 697)			External Validation Cohort (n = 2053)		
	Neoplastic lesions (n = 232)	Non-neoplastic lesions (n = 1397)	*p*-value	Neoplastic lesions (n = 99)	Non-neoplastic lesions (n = 598)	*p*-value	Neoplastic lesions (n = 56)	Non-neoplastic lesions (n =1997)	*p*-value
Age, years, n (%)			0.000			0.039			0.000
<50	45 (19.40%)	440 (31.50%)		21 (21.21%)	159 (26.59%)		13 (23.21%)	822 (41.16%)	
50-59	81 (34.91%)	560 (40.09%)		33 (33.33%)	255 (42.64%)		15 (26.79%)	709 (35.50%)	
60-69	77 (33.19%)	302 (21.62%)		35 (35.35%)	146 (24.41%)		12 (21.43%)	342 (17.13%)	
>69	29 (12.50%)	95 (6.80%)		10 (10.10%)	38 (6.35%)		16 (28.57%)	124 (6.21%)	
Sex, n (%)			0.000			0.000			0.001
Female	67 (28.88%)	749 (53.61%)		29 (29.29%)	321 (53.68%)		14 (25.00%)	949 (47.52%)	
Male	165 (71.12%)	648 (46.39%)		70 (70.71%)	277 (46.32%)		42 (75.00%)	1048 (52.48%)	
Ethnic group, n (%)			0.095			0.160			0.047
Han	165 (71.12%)	1068 (76.45%)		80 (80.81%)	440 (73.58%)		54 (96.43%)	1986 (99.45%)	
Others	67 (28.88%)	329 (23.55%)		19 (19.19%)	158 (26.42%)		2 (3.57%)	11 (0.55%)	
Painkiller intake, n (%)			0.627			0.603			0.118
No	171 (73.71%)	1054 (75.45%)		72 (72.73%)	453 (75.75%)		54 (96.43%)	1782 (89.23%)	
Yes	61 (26.29%)	343 (24.55%)		27 (27.27%)	145 (24.25%)		2 (3.57%)	215 (10.77%)	
Antiplatelet agents intake, n (%)			0.320			0.913			0.784
No	203 (87.50%)	1256 (89.91%)		90 (90.91%)	538 (89.97%)		52 (92.86%)	1866 (93.44%)	
Yes	29 (12.50%)	141 (10.09%)		9 (9.09%)	60 (10.03%)		4 (7.14%)	131 (6.56%)	
Sleep quality , n (%)			0.969			0.950			0.667
Adequate	123 (53.02%)	753 (53.90%)		55 (55.56%)	324 (54.18%)		32 (57.14%)	1010 (50.58%)	
Good	78 (33.62%)	461 (33.00%)		31 (31.31%)	189 (31.61%)		16 (28.57%)	662 (33.15%)	
Poor	31 (13.36%)	183 (13.10%)		13 (13.13%)	85 (14.21%)		8 (14.29%)	325 (16.27%)	
Pickled food intake, n (%)			0.562			0.516			0.348
Not frequent	178 (76.72%)	1099 (78.67%)		78 (78.79%)	491 (82.11%)		52 (92.86%)	1901 (95.19%)	
Frequent	54 (23.28%)	298 (21.33%)		21 (21.21%)	107 (17.89%)		4 (7.14%)	96 (4.81%)	
Smoked food intake, n (%)			0.009			0.205			1.000
Not frequent	180 (77.59%)	1183 (84.68%)		81 (81.82%)	521 (87.12%)		56 (100.00%)	1971 (98.70%)	
Frequent	52 (22.41%)	214 (15.32%)		18 (18.18%)	77 (12.88%)		0 (0.00%)	26 (1.30%)	
History of stomach disease, n (%)^**^			0.449			0.673			0.266
No	178 (76.72%)	1106 (79.17%)		82 (82.83%)	481 (80.43%)		40 (71.43%)	1552 (77.72%)	
Yes	54 (23.28%)	291 (20.83%)		17 (17.17%)	117 (19.57%)		16 (28.57%)	445 (22.28%)	
FHGC, n (%)^***^			0.450			0.100			1.000
No	220 (94.83%)	1343 (96.13%)		92 (92.93%)	577 (96.49%)		53 (94.64%)	1883 (94.29%)	
Yes	12 (5.17%)	54 (3.87%)		7 (7.07%)	21 (3.51%)		3 (5.36%)	114 (5.71%)	
Alcohol consumption, n (%)			0.000			0.261			0.512
No	146 (62.93%)	1123 (80.39%)		76 (76.77%)	491 (82.11%)		34 (60.71%)	1106 (55.38%)	
Yes	86 (37.07%)	274 (19.61%)		23 (23.23%)	107 (17.89%)		22 (39.29%)	891 (44.62%)	
Cigarette smoking, n (%)			0.000			0.017			0.016
No	125 (53.88%)	1071 (76.66%)		63 (63.64%)	452 (75.59%)		34 (60.71%)	1513 (75.76%)	
Yes	107 (46.12%)	326 (23.34%)		36 (36.36%)	146 (24.41%)		22 (39.29%)	484 (24.24%)	
Hot food intake, n (%)			0.631			1.000			0.500
Not frequent	168 (72.41%)	1036 (74.16%)		74 (74.75%)	444 (74.25%)		46 (82.14%)	1705 (85.38%)	
Frequent	64 (27.59%)	361 (25.84%)		25 (25.25%)	154 (25.75%)		10 (17.86%)	292 (14.62%)	
Leftover food intake, n (%)			0.019			0.136			0.003
Not frequent	167 (71.98%)	1105 (79.10%)		70 (70.71%)	467 (78.09%)		45 (80.36%)	1850 (92.64%)	
Frequent	65 (28.02%)	292 (20.90%)		29 (29.29%)	131 (21.91%)		11 (19.64%)	147 (7.36%)	
Mealtime, n (%)			0.796			0.471			0.140
Unregular	18 (7.76%)	119 (8.52%)		9 (9.09%)	39 (6.52%)		6 (10.71%)	115 (5.76%)	
Regular	214 (92.24%)	1278 (91.48%)		90 (90.91%)	559 (93.48%)		50 (89.29%)	1882 (94.24%)	
Overeat and overdrink, n (%)			0.456			1.000			0.339
Not frequent	226 (97.41%)	1370 (98.07%)		97 (97.98%)	584 (97.66%)		54 (96.43%)	1955 (97.90%)	
Frequent	6 (2.59%)	27 (1.93%)		2 (2.02%)	14 (2.34%)		2 (3.57%)	42 (2.10%)	
Fresh fruit intake, n (%)			0.713			0.089			0.964
Not frequent	150 (64.66%)	924 (66.14%)		75 (75.76%)	398 (66.56%)		27 (48.21%)	969 (48.52%)	
Frequent	82 (35.34%)	473 (33.86%)		24 (24.24%)	200 (33.44%)		29 (51.79%)	1028 (51.48%)	
Fresh vegetables intake, n (%)			0.047			0.421			0.625
Not frequent	65 (28.02%)	488 (34.93%)		40 (40.40%)	213 (35.62%)		7 (12.50%)	209 (10.47%)	
Frequent	167 (71.98%)	909 (65.07%)		59 (59.60%)	385 (64.38%)		49 (87.50%)	1788 (89.53%)	
Red meat intake, n (%)^#^			0.113			0.607			0.463
Not frequent	130 (56.03%)	863 (61.78%)		60 (60.61%)	382 (63.88%)		15 (26.79%)	627 (31.40%)	
Frequent	102 (43.97%)	534 (38.22%)		39 (39.39%)	216 (36.12%)		41 (73.21%)	1370 (68.60%)	
Educational level, n (%)			0.026			0.921			0.032
illiteracy	35 (15.09%)	139 (9.95%)		10 (10.10%)	55 (9.20%)		5 (8.93%)	61 (3.05%)	
literacy	197 (84.91%)	1258 (90.05%)		89 (89.90%)	543 (90.80%)		51 (91.07%)	1936 (96.95%)	
Digestive tract symptom, n (%)^##^			0.503			0.913			0.168
No	107 (46.12%)	681 (48.75%)		48 (48.48%)	297 (49.67%)		13 (23.21%)	637 (31.90%)	
Yes	125 (53.88%)	716 (51.25%)		51 (51.52%)	301 (50.33%)		43 (76.79%)	1360 (68.10%)	
Type of residential area, n (%)			0.764			0.518			0.005
Rural	210 (90.52%)	1252 (89.62%)		92 (92.93%)	540 (90.30%)		16 (28.57%)	299 (14.97%)	
Urban	22 (9.48%)	145 (10.38%)		7 (7.07%)	58 (9.70%)		40 (71.43%)	1698 (85.03%)	
Occupation, n (%)			0.599			0.071			0.012
farmer	184 (79.31%)	1132 (81.03%)		88 (88.89%)	483 (80.77%)		10 (17.86%)	156 (7.81%)	
unfarmer	48 (20.69%)	265 (18.97%)		11 (11.11%)	115 (19.23%)		46 (82.14%)	1841 (92.19%)	
Income, n (%)			1.000			0.559			0.705
High	48 (20.69%)	286 (20.47%)		19 (19.19%)	134 (22.41%)		31 (55.36%)	1156 (57.89%)	
Low	184 (79.31%)	1111 (79.53%)		80 (80.81%)	464 (77.59%)		25 (44.64%)	841 (42.11%)	
Marital status, n (%)^###^			1.000			0.138			1.000
Married	212 (91.38%)	1278 (91.48%)		87 (87.88%)	555 (92.81%)		51 (91.07%)	1813 (90.79%)	
Unmarried	20 (8.62%)	119 (8.52%)		12 (12.12%)	43 (7.19%)		5 (8.93%)	184 (9.21%)	
Current and Past Manual labour, n (%)			0.462			1.000			0.115
No	30 (12.93%)	210 (15.03%)		12 (12.12%)	71 (11.87%)		35 (62.50%)	1440 (72.11%)	
Yes	202 (87.07%)	1187 (84.97%)		87 (87.88%)	527 (88.13%)		21 (37.50%)	557 (27.89%)	
BMI (kg/m^2), n (%)^§^			0.071			1.000			0.005
<18.5	32 (13.79%)	1097 (78.53%)		9 (9.09%)	52 (8.70%)		7 (12.50%)	72 (3.61%)	
>=18.5	200 (86.21%)	300 (21.47%)		90 (90.91%)	546 (91.30%)		49 (87.50%)	1925 (96.39%)	
Hp, n (%)			0.159			0.222			1.000
Negative	212 (91.38%)	1314 (94.06%)		89 (89.90%)	561 (93.81%)		42 (75.00%)	1510 (75.61%)	
Positive	20 (8.62%)	83 (5.94%)		10 (10.10%)	37 (6.19%)		14 (25.00%)	487 (24.39%)	
Water intake (ml/day), n (%)			0.2817			0.9399			0.590
>1000	39 (16.81%)	194 (13.89%)		16 (16.16%)	102 (17.06%)		15 (26.79%)	601 (30.10%)	
≤1000	193 (83.19%)	1203 (86.11%)		83 (83.84%)	496 (82.94%)		41 (73.21%)	1936 (96.95%)	
Chronic disease, n (%)			0.2385			0.6919			0.504
No	156 (67.24%)	996 (71.30%)		74 (74.75%)	432 (72.24%)		33 (58.93%)	1264 (63.29%)	
Yes	76 (32.76%)	401 (28.70%)		25 (25.25%)	166 (27.76%)		23 (41.07%)	733 (36.71%)	

Data are presented as n for categorical variables, mean (SD) for continuous variables. For variables about eating habits, two categories for frequency of consumption were provided, that is, not frequent (<3 times/week) and frequent (at least 3 times/week).

*Variables were questionnaire-based predictors which met any one of the criteria below: 1) biologic plausibility in predicting the risk of GC or 2) reported in at least two published risk prediction studies.

**History of stomach diseases includes gastritis, gastric ulcers, hypertrophic gastritis, remnant stomach and gastric polyps.

***Family history of gastric cancer (FHGC) is defined as GC cases among first-degree relatives.

^#^Red meat includes beef, pork and lamb.

^##^Digestive tract symptom includes heartburn, problems swallowing or passing food along your esophagus and stomach, stomach pain, nausea, and vomiting.

^###^Marital status classifies as married and unmarried (including single, separated, divorced, widowed, and unmarried patients).

^§^Body mass index (BMI): weight (kg)/height (m)^2^.

*p values refer to comparison between low-risk participants and high-risk participants in the univariate analysis.

p values refer to comparison between neoplastic lesions and non-neoplastic lesions in the univariate analysis.

**Table 2:** Characteristics of the training cohort and the internal validation cohort.

Variables	Total participants (n = 2326)	Training Cohort (n = 1629)	Validation Cohort (n = 697)	*p*-value
Age, years, n (%)				0.174
<50	665 (28.59%)	485 (29.77%)	180 (25.82%)	
50-59	929 (39.94%)	641 (39.35%)	288 (41.32%)	
60-69	560 (24.07%)	379 (23.27%)	181 (25.97%)	
>69	172 (7.40%)	124 (7.61%)	48 (6.89%)	
Sex, n (%)				0.993
Female	1166 (50.13%)	816 (50.09%)	350 (50.22%)	
Male	1160 (49.87%)	813 (49.91%)	347 (49.78%)	
Ethnic group, n (%)				0.614
Han	1753 (75.37%)	1233 (75.69%)	520 (74.61%)	
Others	573 (24.63%)	396 (24.31%)	177 (25.39%)	
Painkiller intake, n (%)				0.992
No	1750 (75.24%)	1225 (75.20%)	525 (75.32%)	
Yes	576 (24.76%)	404 (24.80%)	172 (24.68%)	
Antiplatelet agents intake, n (%)				0.752
No	2087 (89.72%)	1459 (89.56%)	628 (90.10%)	
Yes	239 (10.28%)	170 (10.44%)	69 (9.90%)	
Sleep quality, n (%)				0.708
Adequate	1255 (53.96%)	876 (53.77%)	379 (54.38%)	
Good	759 (32.63%)	539 (33.09%)	220 (31.56%)	
Poor	312 (13.41%)	214 (13.14%)	98 (14.06%)	
Pickled food intake, n (%)				0.086
Not frequent	1846 (79.36%)	1277 (78.39%)	569 (81.64%)	
Frequent	480 (20.64%)	352 (21.61%)	128 (18.36%)	
Smoked food intake, n (%)				0.113
Not frequent	1965 (84.48%)	1363 (83.67%)	602 (86.37%)	
Frequent	361 (15.52%)	266 (16.33%)	95 (13.63%)	
History of stomach disease, n (%)				0.312
No	1847 (79.41%)	1284 (78.82%)	563 (80.77%)	
Yes	479 (20.59%)	345 (21.18%)	134 (19.23%)	
FHGC, n (%)				1.000
No	2232 (95.96%)	1563 (95.95%)	669 (95.98%)	
Yes	94 (4.04%)	66 (4.05%)	28 (4.02%)	
Alcohol consumption, n (%)				0.070
No	1836 (78.93%)	1269 (77.90%)	567 (81.35%)	
Yes	490 (21.07%)	360 (22.10%)	130 (18.65%)	
Cigarette smoking, n (%)				0.854
No	1711 (73.56%)	1196 (73.42%)	515 (73.89%)	
Yes	615 (26.44%)	433 (26.58%)	182 (26.11%)	
Hot food intake, n (%)				0.878
Not frequent	1722 (74.03%)	1204 (73.91%)	518 (74.32%)	
Frequent	604 (25.97%)	425 (26.09%)	179 (25.68%)	
Leftover food intake, n (%)				0.618
Not frequent	1809 (77.77%)	1272 (78.08%)	537 (77.04%)	
Frequent	517 (22.23%)	357 (21.92%)	160 (22.96%)	
Mealtime, n (%)				0.246
Unregular	185 (7.95%)	137 (8.41%)	48 (6.89%)	
Regular	2141 (92.05%)	1492 (91.59%)	649 (93.11%)	
Overeat and overdrink, n (%)				0.797
Not frequent	2277 (97.89%)	1596 (97.97%)	681 (97.70%)	
Frequent	49 (2.11%)	33 (2.03%)	16 (2.30%)	
Fresh fruit intake, n (%)				0.392
Not frequent	1547 (66.51%)	1074 (65.93%)	473 (67.86%)	
Frequent	779 (33.49%)	555 (34.07%)	224 (32.14%)	
Fresh vegetables intake, n (%)				0.296
Not frequent	806 (34.65%)	553 (33.95%)	253 (36.30%)	
Frequent	1520 (65.35%)	1076 (66.05%)	444 (63.70%)	
Red meat intake, n (%)				0.285
Not frequent	1435 (61.69%)	993 (60.96%)	442 (63.41%)	
Frequent	891 (38.31%)	636 (39.04%)	255 (36.59%)	
Educational level, n (%)				0.362
illiteracy	239 (10.28%)	174 (10.68%)	65 (9.33%)	
literacy	2087 (89.72%)	1455 (89.32%)	632 (90.67%)	
Digestive tract symptom, n (%)				0.651
No	1133 (48.71%)	788 (48.37%)	345 (49.50%)	
Yes	1193 (51.29%)	841 (51.63%)	352 (50.50%)	
Type of residential area, n (%)				0.544
Rural	2094 (90.03%)	1462 (89.75%)	632 (90.67%)	
Urban	232 (9.97%)	167 (10.25%)	65 (9.33%)	
Occupation, n (%)				0.559
farmer	1887 (81.13%)	1316 (80.79%)	571 (81.92%)	
unfarmer	439 (18.87%)	313 (19.21%)	126 (18.08%)	
Income, n (%)				0.465
High	487 (20.94%)	334 (20.50%)	153 (21.95%)	
Low	1839 (79.06%)	1295 (79.50%)	544 (78.05%)	
Marital status, n (%)				0.666
Married	2132 (91.66%)	1490 (91.47%)	642 (92.11%)	
Unmarried	194 (8.34%)	139 (8.53%)	55 (7.89%)	
Current or past manual labour, n (%)				0.082
No	323 (13.89%)	240 (14.73%)	83 (11.91%)	
Yes	2003 (86.11%)	1389 (85.27%)	614 (88.09%)	
BMI (kg/m^2), n (%)				0.299
<18.5	228 (9.80%)	167 (10.25%)	61 (8.75%)	
≥18.5	2098 (90.20%)	1462 (89.75%)	636 (91.25%)	
Hp, n (%)				0.775
Negative	2176 (93.55%)	1526 (93.68%)	650 (93.26%)	
Positive	150 (6.45%)	103 (6.32%)	47 (6.74%)	
Water intake (ml/day), n (%)				0.1193
>1000	351 (15.09%)	233 (14.30%)	118 (16.93%)	
≤1000	1975 (84.91%)	1396 (85.70%)	579 (83.07%)	
Chronic disease, n (%)				0.3858
No	1658 (71.28%)	1152 (70.72%)	506 (72.60%)	
Yes	668 (28.72%)	477 (29.28%)	191 (27.40%)	

p values refer to comparison between training cohort and the validation cohort in the univariate analysis.

### Prediction factor identification

**Figure 2:**
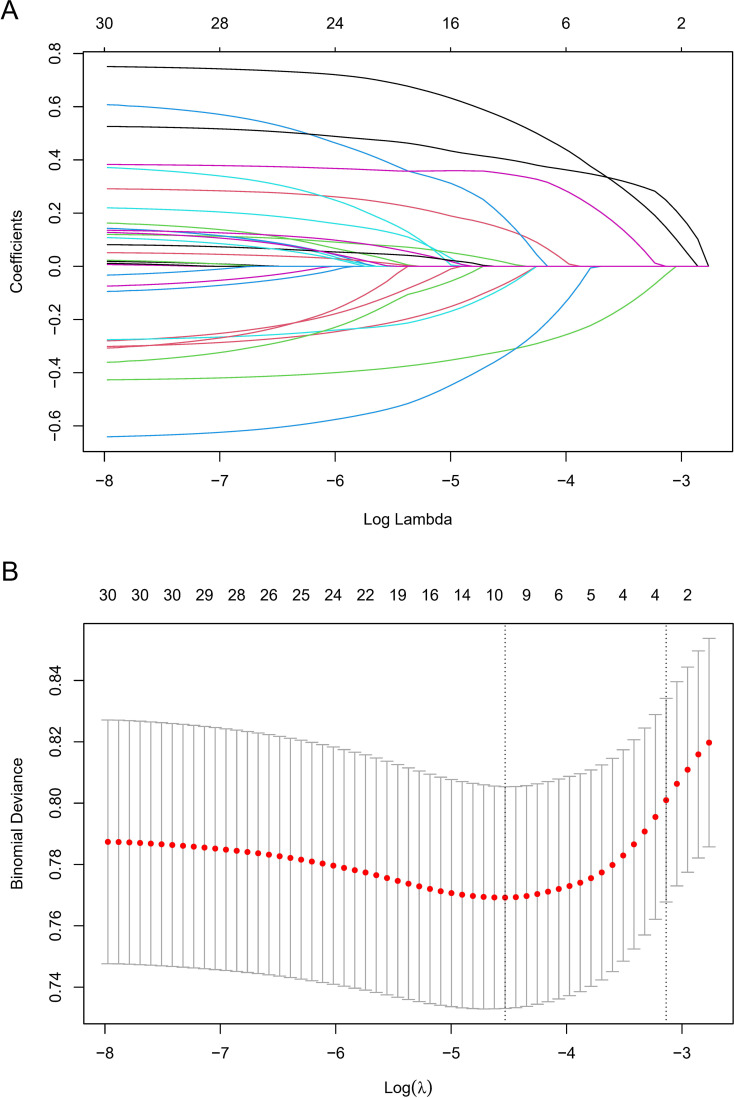
Variable selection by LASSO regression model. **(A)** A coefficient profile plot was produced against the log(lambda) sequence. The upper Abscissa is the number of non-zero coefficients in this model, and the ordinate is the coefficient value. **(B)** The optimal penalization coefficient (lambda) in the LASSO model was identified by 10-fold cross-validation via minimum criterion. The left vertical dotted line represents the minimum criterion, and the right line represents the cross-validated error within one standard error of the minimum. The upper Abscissa indicates the number of independent variables that still exist in the model. In the present study, predictor selection was performed according to the minimum criterion. LASSO coefficient profiles of the 30 factors to be screened. The vertical line on the left indicates that the optimal lambda results in the 10 features with non-zero coefficients.

To identify potentially significant indicators associated with GC development, a LASSO regression algorithm was applied to the training cohort. The age stratification according to published papers (6, 21). The variation characteristics of the coefficient of these variables were shown in **Figure 2A**. The most appropriate tuning parameter lambda for the LASSO regression was 0.01075537 when the partial likelihood binomial deviance reached its minimum value. After LASSO regression analysis, 10 variables remained significant predictors of neoplastic lesions, including sex, ethnic groups, age, smoked food consumption, alcohol consumption, cigarette smoking, leftover food consumption, education level, BMI, and Hp infection status (**Figure 2**).

These 10 variables were further selected as candidate variables for multivariable logistic analysis. Six variables, including age, sex, alcohol consumption, cigarette smoking, education level, and Hp infection status, were independent predictors for the development of neoplastic lesions after multivariable selection (**Table 3**).

**Table 3:** Multivariable analysis of risk factors for developing neoplastic lesions.

	Multivariable analysis			
Variables	OR	95%CI lower	95% CI Upper	*p*-value
Covariates				
Age, years				
<50	1 (ref.)			
50-59	1.46	0.99	2.20	0.061
60-69	2.54	1.68	3.88	0.000
>69	2.96	1.71	5.09	0.000
Sex				
Female	1 (ref.)			
Male	2.14	1.46	3.14	0.000
Ethnic group				
Han	1 (ref.)			
Others	1.32	0.95	1.83	0.091
Smoked food intake				
Not frequent	1 (ref.)			
Frequent	1.31	0.90	1.89	0.157
Alcohol consumption				
No	1 (ref.)			
Yes	1.50	1.04	2.16	0.028
Cigarette smoking				
No	1 (ref.)			
Yes	1.62	1.10	2.40	0.014
Leftover food intake				
Not frequent	1 (ref.)			
Frequent	1.11	0.78	1.57	0.542
Educational level				
illiteracy	1 (ref.)			
literacy	0.55	0.36	0.87	0.008
BMI (kg/m^2)				
<18.5	1 (ref.)			
≥18.5	0.72	0.47	1.13	0.140
Hp				
Negative	1 (ref.)			
Positive	1.81	1.02	3.11	0.036

OR, odds ratio; CI, Confidence Interval.

### Nomogram construction

**Figure 3:**
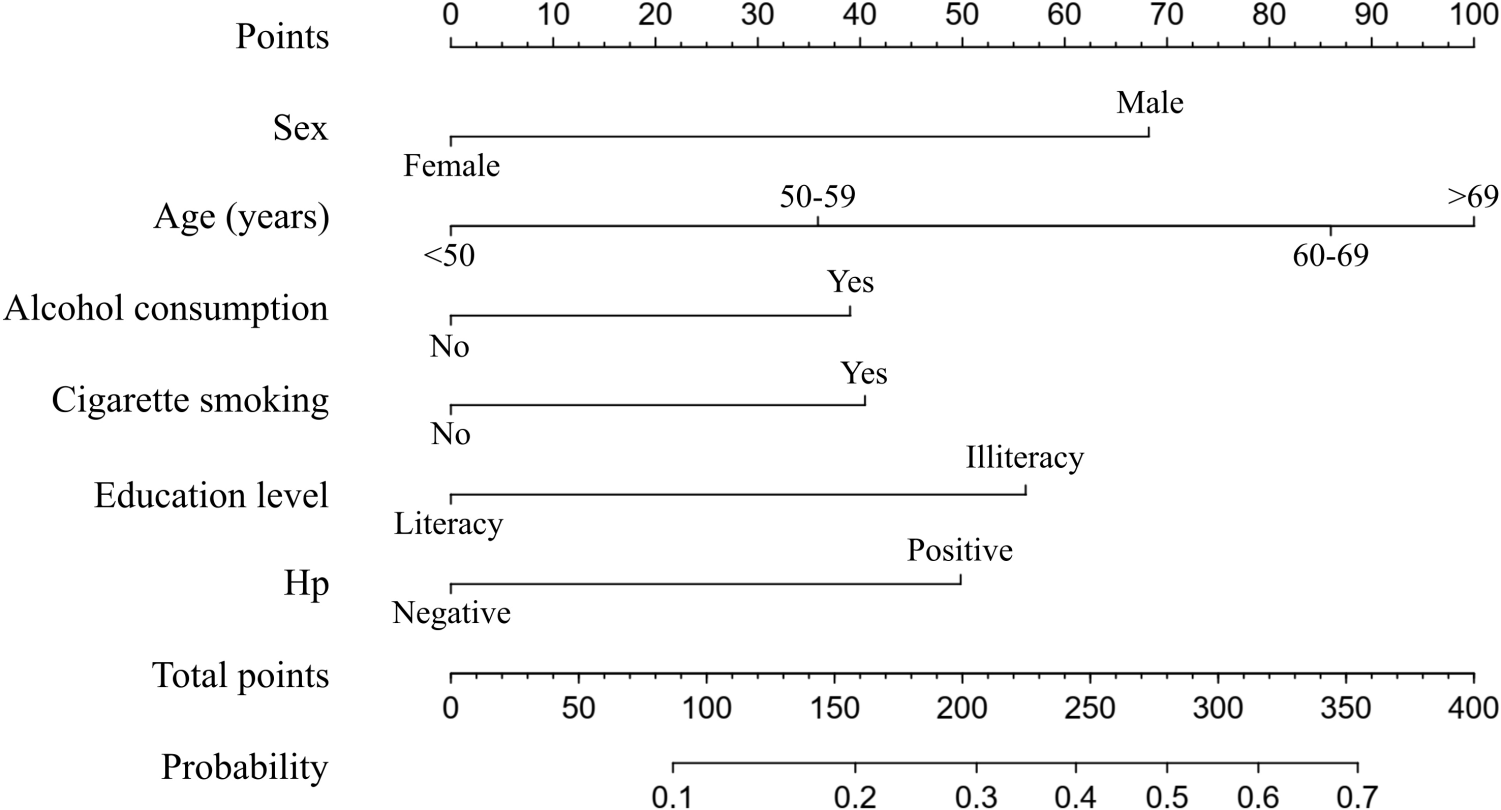
A nomogram predicting the probability of developing neoplastic lesions for individual participant. The scores of each variable are added to obtain the total score, and then a vertical line is subtracted from the row of total-points to estimate the probability of developing neoplastic lesions. The neoplastic lesions incidence risk nomogram was developed in the array, with sex, age, alcohol consumption, cigarette smoking, education level, and Hp infection status incorporated.

Next, the nomogram used to predict the probabilities of developing neoplastic lesions was formulated using significant independent factors, including age, sex, alcohol consumption, cigarette smoking, education level, and Hp infection status. For an individual participant, the first row shows the points ranging from 0 to 100 received for each variable value which is loaded on each variable axis (row 2-7), and the total score was calculated by summing the individual scores (row 8). The individual probabilities of developing neoplastic lesions were also obtained from the nomogram (**Figure 3**).

### Assessment of predictive accuracy of the nomogram

**Figure 4:**
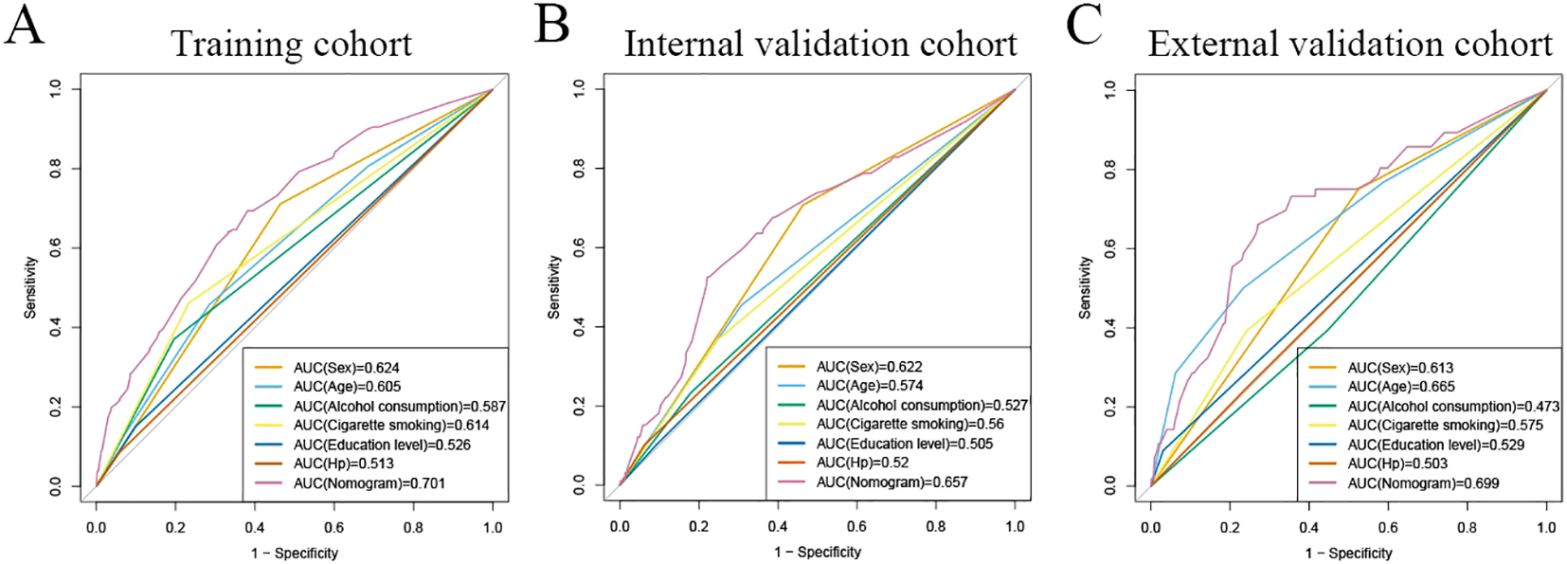
ROC curves for predicting risk of developing neoplastic lesions. A receiver operating characteristics (ROC) curve of the nomogram model and single independent predictor in the training cohort **(A)**, internal validation cohort **(B)**, and external validation cohort **(C)**. The model displayed reliable diagnostic performance for prediction of developing neoplastic lesions in both the training cohort (AUC, 0.701), internal validation cohort (AUC, 0.657), and external validation cohort (AUC, 0.699).

**Figure 5:**
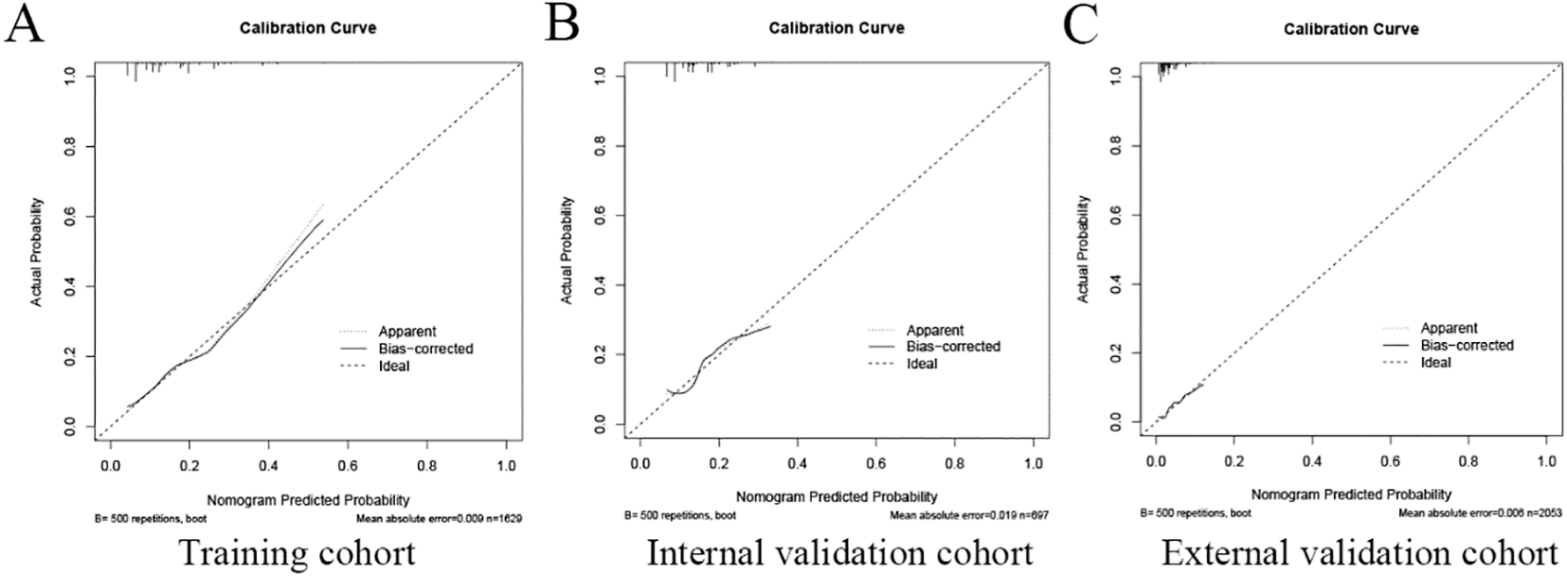
The calibration plot of the nomogram in the training and validation cohort. The calibration plot was applied to compare the agreement between actual and predicted probability of developing neoplastic lesions in the training cohort **(A)**, internal validation cohort **(B)**, and external validation cohort **(C)**.

The area under the operating characteristic curve (ROC) curve (AUC) values were analyzed to investigate the discrimination of the nomogram, which was 0.701 (95% CI, 0.665-0.738) in the training cohort and 0.657 (95% CI, 0.596-0.717) in the internal validation cohort, and 0.699 (95% CI, 0.625-0.773) in the external validation cohort, respectively (**Figure 4**), indicating that the model has good predictive ability. The calibration curves were also applied to verify the predicted effect of the nomogram, and the calibration plot was highly consistent between the predicted and observed severity in the training (**Figure 5A**), the internal validation (**Figure 5B**), and the external validation cohorts (**Figure 5C**). The DeLong test results indicated that the predictive performance of the nomogram model was significantly higher than that of the single independent predictor in the training cohort, internal validation cohort and external validation cohort, but there was no significant difference between nomogram model and age in the validation cohort (**Table 4**).

**Table 4:** Delong test between nomogram model and single independent predictor.

Model	*p*-value		
	Training cohort	Internal validation cohort	External validation cohort
Nomogram vs age	0.0000	0.0036	0.4184
Nomogram vs sex	0.0000	0.0856	0.0006
Nomogram vs alcohol consumption	0.0000	0.0000	0.0000
Nomogram vs cigarette smoking	0.0000	0.0003	0.0007
Nomogram vs educational level	0.0000	0.0000	0.0000
Nomogram vs hp	0.0000	0.0000	0.0000

### Clinical application value of nomogram

**Figure 6:**
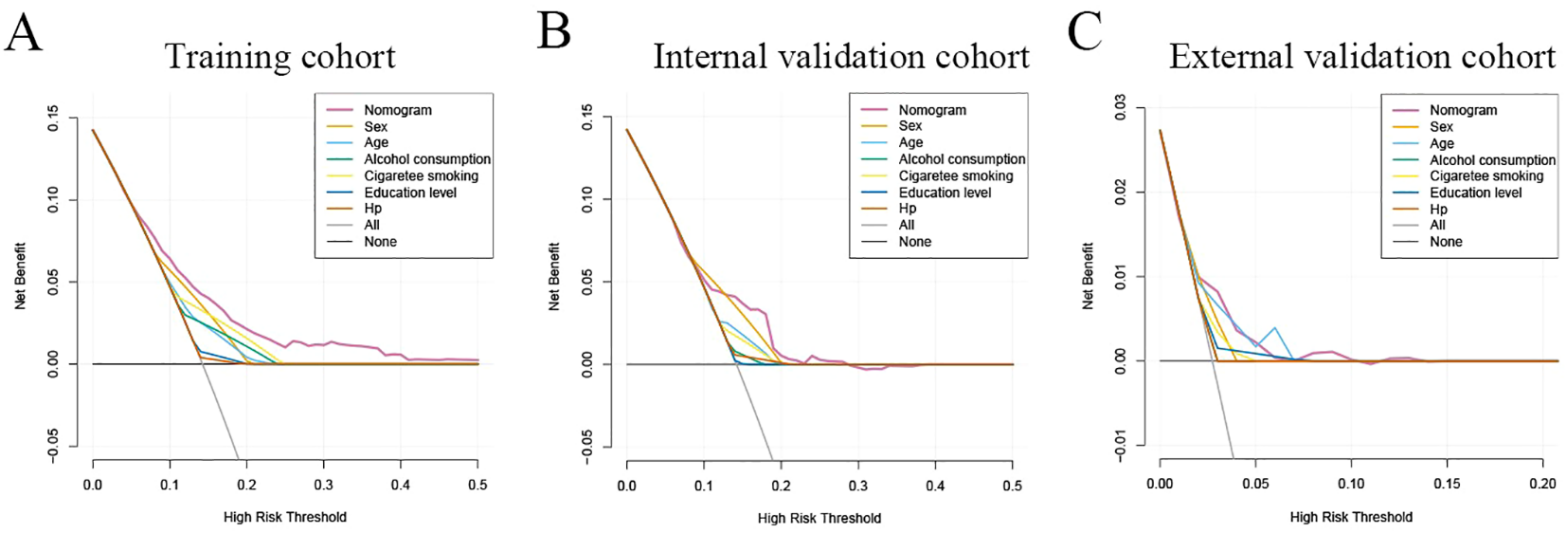
Clinical practice of the nomogram. Decision curve analysis (DCA) for the prediction nomogram model and single independent predictor in the training cohort **(A)**, internal validation cohort **(B)**, and external validation cohort **(C)**. The y-axis measures the net benefit, x-axis indicates threshold probability. The red solid line represents the combined nomogram.

The decision curve analysis (DCA) analysis was performed to assess the clinical benefit of the model. As shown in **Figure 6**, the abscissa represents the threshold probability, while the ordinate depicts the net benefit rate after subtracting the pros and cons. The two straight lines in the figure illustrate the two extreme cases. The horizontal line indicates that all participants are considered to be free of neoplastic lesions, thereby indicating a net benefit of 0 in the absence of intervention. The slash denotes the net benefit when all participants are considered to have neoplastic lesions, and all receive the intervention. Our nomogram showed a net benefit across a wide range of threshold probabilities for predicting the risk of developing neoplastic lesions and the nomogram model showed better clinical utility than the predictive value of any single variable, regardless of whether the training, internal validation cohort or external validation cohort was used (**Figure 6**).

## Discussion

In this study, we established a nomogram model to predict the risk of GC developing using the training cohort and achieved good performance in the training, internal validation, and external validation cohorts. Our finding revealed that advanced age, male sex, alcohol consumption, cigarette smoking, illiteracy, and Hp infection were identified as risk factors that increased the risk of developing GC. Both the calibration curve and the ROC curve indicate that the nomogram has a high accuracy and has a good discrimination. In addition, the DCA shows that the nomogram has good clinical utility. Therefore, this nomogram can serve as a simple, accuracy, and convenient prescreening tool to predict the individual risk of developing GC in China.

A comprehensive understanding of the risk factors for developing GC is essential to identify individuals at high risk who require endoscopic examination. Our multivariable analysis revealed that age, sex, alcohol consumption, cigarette smoking, education level, and Hp infection status were independent predictors of neoplastic lesions (**Table 3**), which is consistent with findings from previous studies (6, 22–27). It indicates that the predictive factors our selected were reliable. As noted in previous research, the incidence of GC has been shown to increase with age, particularly after 40 years old (6, 22, 23, 28). Consistent with these studies, our results indicated that advanced age was associated with a higher risk of neoplastic lesions developing. Previous studies have shown that men have a higher risk of developing GC than women, suggesting sex also plays an important role in the development of GC (25, 28, 29). This is consistent with the conclusions we have obtained, that male sex has an increased risk for neoplastic lesions. Education level was also significant factor in the prediction model, consistent with the previous meta-analysis (30). In contrast, we did not find significant associations between income level, occupation, and the risk of neoplastic lesions. This is consistent with previous studies (22, 31). Lifestyle factors have been extensively studied in relation to GC risk (32, 33). Unhealthy lifestyles, including cigarette smoking and alcohol consumption, have previously been reported to be associated with a higher risk of GC (26, 34–37). Consistent with these studies, our results indicated that alcohol consumption and cigarette smoking were associated with an increased risk of developing neoplastic lesions. FHGC is well accepted as an important risk factor of GC development (38–40). However, our present study showed that it was weakly correlated with risk of neoplastic lesions (**Table 1**). This finding can be explained by the low prevalence of FHGC-related neoplastic lesions observed in our study participants. Additionally, the neoplastic lesion group included GIN, which was not considered to be significantly associated with FHGC (41, 42). Conversely, some studies have indicated that there is no positive correlation between FHGC and GC in the Chinese population (22, 43).

Hp infection has been shown to be the most prominent factor for the development of GC (6, 27, 44). More than 90% of GC patients have current or past Hp infection (45, 46). Since the discovery of Hp, numerous studies have confirmed the association between Hp and GC (22, 27, 47). Consistent with previous studies, our results show that Hp infection increases the risk of developing neoplastic lesions. It has been recommended to eradicate Hp in patients with CAG and IM by the guidelines in Asia and Europe (48), and the eradication of Hp infection is associated with a reduced incidence of GC (49, 50). Therefore, Hp eradication treatment could be used as an important strategy for GC prevention in high-risk populations.

This study has several strengths. Firstly, the utilization of questionnaire data to develop the risk prediction model increases the feasibility of implementing it in real-world screening programs, particularly in areas with inconvenient transport access such as plateau-mountain regions. This approach provides a practical and cost-effective means of identifying individuals at risk of developing GC. Secondly, this newly proposed risk prediction tool has included GIN, which helps to accurately identify individuals with such lesions to further improve their interception and thus potentially reduce the incidence of GC. Third, the performance of this new prediction model was validated in both internally and externally clinical outpatient cohorts with heterogeneous characteristics. Therefore, this model is believed to have a good generalizability and applicability in the Chinese population. Forth, the stability of the Fengqing’s cohort is another strength of the study. The establishment of a stable cohort ensures long-term follow-up of participants, allowing valuable data to be collected over an extended period of time. In addition, the ability to update the cohort in a timely manner allows new information and finding to be incorporated into future analyses, improving the accuracy and relevance of the risk prediction model.

Several limitations of this study need to be noted regarding. First, the number of GC patients in the current cohort we used was relatively small, which may result in the missing of crucial demographic factors about GC development. Further research, particularly in areas with a high incidence of GC, is needed to validate the results and ensure their applicability to different populations. Second, the participants enrolled in our study were from clinical outpatient, which might induce selection bias, even though the study was stringently designed. Future research including the general population is essential to externally validate the results and increase the applicability of the prediction model. Third, the effect of risk factors for neoplastic lesions might differ between men and women. We performed sex-specific analysis, however, after LASSO regression followed by multivariable logistic analysis, none factor was significant in female participants because of the small number of female cases in neoplastic lesions (**Supplementary Table 1**). Future studies are warranted to clarify the risk factors of neoplastic lesions in different sex. Forth, in order for the prediction tool to be widely accepted and used in practice, only a limited number of readily available lifestyle factors were considered. While this increases the feasibility and simplicity of the tool, it may overlook other potential risk factors that may contribute to the development of GC. Further research incorporating a wider range of risk factors, including genetic, and environmental, may provide a more comprehensive understanding of GC risk prediction. In the future, it is expected to combine a wider range of risk factors, such as serological test, plasma metabolite profiling, and genetic factors, with machine learning or deep learning to create high-accuracy prediction models for GC.

## Conclusions

In conclusion, we have developed a low-cost, easy-to-use, and powerful prediction model. The nomogram model developed in this study demonstrated the ability to accurately predict the likelihood of neoplastic lesions in China. This model could serve as a powerful and practical application tool prior to gastroscopy.

## Data Availability

The raw data supporting the conclusions of this article will be made available by the authors, without undue reservation.
