# Supplementary material for: Development and validation of a nomogram for predicting the risk of developing gastric cancer based on a questionnaire: a cross–sectional study
Source: Front Oncol. 2024 Nov 11;14:1351967. doi: 10.3389/fonc.2024.1351967 (PMC11586234; doi:10.3389/fonc.2024.1351967)

Supplementary


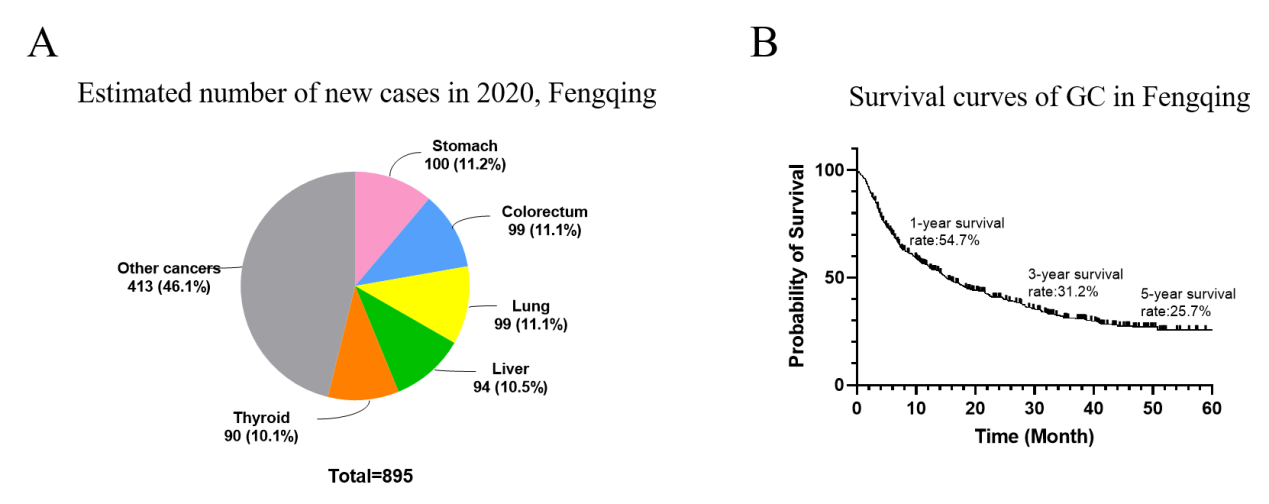


Figure S1. The epidemic status of GC in Fengqing County. (A) Five leading cancer types in the estimated new cancer cases, Fengqing County, 2020. (B) Kaplan–Meier curve, 5-year survival rate of GC in Fengqing County.

Table 1. Multivariable analysis of risk factors for developing neoplastic lesions in female.


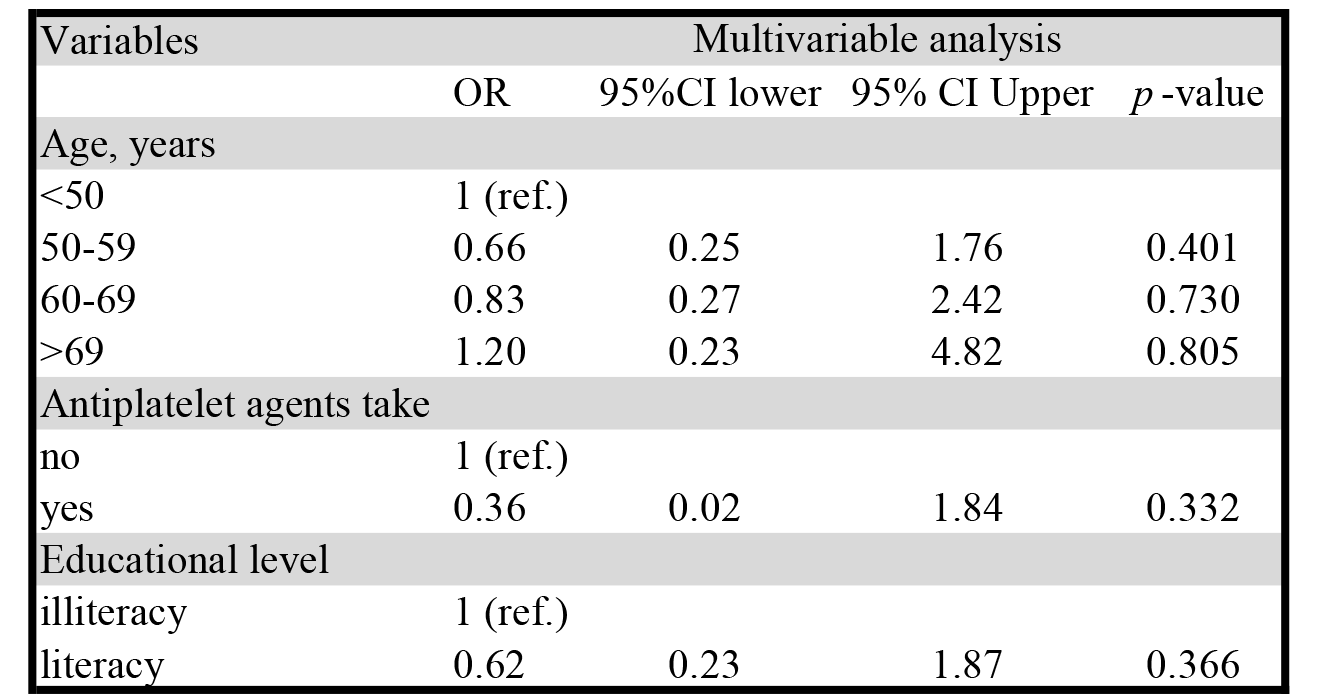

Supplement: Supplementary file 1 [file DataSheet1.docx]
